# Factors associated with tick bites and pathogen prevalence in ticks parasitizing humans in Georgia, USA

**DOI:** 10.1186/s13071-016-1408-6

**Published:** 2016-03-02

**Authors:** Elizabeth R. Gleim, Laurel E. Garrison, Marianne S. Vello, Mason Y. Savage, Gaylord Lopez, Roy D. Berghaus, Michael J. Yabsley

**Affiliations:** Southeastern Cooperative Wildlife Disease Study, College of Veterinary Medicine, The University of Georgia, 589 D.W. Brooks Dr., Wildlife Health Bldg, Athens, GA 30602 USA; Warnell School of Forestry and Natural Resources, The University of Georgia, 180 E. Green St., Athens, GA 30602 USA; Current Address: Oxford College of Emory University, 150 Few Cr, Oxford, GA 30054 USA; Georgia Department of Human Resources, Division of Public Health, 2 Peachtree St. NW, Atlanta, GA 30303 USA; Current address: Centers for Disease Control and Prevention, 1600 Clifton Rd. NE, MS C-25, Atlanta, GA 30333 USA; Current address: College of Veterinary Medicine, North Carolina State University, 1052 William Moore Dr, Raleigh, NC 27606 USA; Georgia Poison Center, 80 Jesse Hill Junior Dr. SE, Atlanta, GA 30303 USA; Department of Population Health, College of Veterinary Medicine, The University of Georgia, 501 D.W. Brooks Dr, Atlanta, GA 30602 USA

**Keywords:** Epidemiology, Tick-borne pathogens, Tick-borne disease, Rickettsia, Ticks, Georgia

## Abstract

**Background:**

The incidence and emergence of tick-borne diseases has increased dramatically in the United States during the past 30 years, yet few large-scale epidemiological studies have been performed on individuals bitten by ticks. Epidemiological information, including disease development, may provide valuable information regarding effectiveness of tick bite prevention education, pathogen transmission, human-disease dynamics, and potential implications for under reporting of tick-borne diseases.

**Methods:**

Ticks found attached to Georgia residents were submitted for identification and polymerase chain reaction (PCR) testing for *Francisella tularensis*, *Ehrlichia*, *Anaplasma*, *Borrelia*, and *Rickettsia* spp. Tick bite victims were interviewed three weeks after the tick bite to identify various epidemiologic factors associated with infestation and if signs suggestive of a tick-borne disease had developed. Fisher’s exact test of independence was used to evaluate associations between various factors evaluated in the study. A multivariable logistic regression model was used for the prediction of non-specific illness post-tick bite.

**Results:**

From April 2005-December 2006, 444 participants submitted 597 ticks (426 *Amblyomma americanum*, 142 *Dermacentor variabilis*, 19 *A. maculatum*, 7 *Ixodes scapularis*, 3 *Amblyomma* sp.) which originated from 95 counties. Only 25 (34 %) of 74 interviewed individuals purposely took tick bite prevention measures. Ticks that were PCR positive for bacterial organisms were attached to 136 participants. Of the 77 participants who developed non-specific illness, 50 did not have PCR positive ticks, whereas 27 did have PCR positive tick (s). Of those 27 individuals, 12 fit the criteria for a possible tick-borne illness (i.e., tick attached >6 h [if known], ≥4 day incubation period, and the individual exhibited clinical symptoms typical of a tick-borne illness without exhibiting cough, sore throat, or sinus congestion). Ticks from these individuals were positive for *R. amblyommii* (*n* = 8), *E. ewingii* (*n* = *1*), *R. montana* (*n* = 1), *R. rhiphicephali* (*n* = 1), and *Rickettsia* sp. TR-39 (*n* = 1).

**Conclusions:**

Although illnesses reported in this study cannot definitively be connected with tick bites, it does provide insight into development, diagnosis, and treatment of possible tick-borne diseases post-tick bite. The study also provided data on pathogen prevalence, and epidemiologic factors associated with tick bites, as well as tick presence by county in Georgia.

## Background

In the United States, at least 11 species of ticks are vectors of pathogens of public health importance. Of these, two species (*Amblyomma americanum* and *Ixodes scapularis*) are responsible for the transmission of most known pathogens. For example, *A. americanum* transmits the causative agents of human monocytic ehrlichiosis (HME), *Ehrlichia ewingii* ehrlichiosis, Panola Mountain ehrlichiosis, tularemia, southern tick-associated rash illness (STARI) and the newly identified heartland virus [1]. Additionally, *I. scapularis* transmits the causative agents of Lyme disease, *Borrelia miyamotoi* relapsing fever, human granulocytic anaplasmosis (HGA), and babesiosis. Other tick-borne diseases of importance, particularly in the Southeast, include Rocky Mountain spotted fever (RMSF) and *Rickettsia parkeri* rickettsiosis.

While most of these tick-borne diseases are nationally notifiable to the Centers for Disease Control and Prevention, it is generally thought that tick-borne diseases are under reported due to misdiagnosis or failure to seek medical treatment, report disease, or identify specific disease agents [2–4]. Furthermore, demographic and epidemiological information is infrequently collected on these patients which could prove to be useful in targeting high risk groups or behaviors [5–10]. A single study in which epidemiologic and demographic information was collected from tick-bite victims was performed in Kentucky [11]. However, there was a small sample size (*n* = 33) and study participants were geographically and demographically restricted to individuals eligible for health services at a single military base.

Additionally, because being bitten by an infected tick does not necessarily result in transmission of the pathogen and those who do develop disease are not always reported, information on human encounter rates with infected ticks is nearly impossible to obtain. However, such data could assist in identifying geographic regions with high disease risk, as well as provide insights into pathogen transmission dynamics. While several studies have been published regarding ticks parasitizing humans in the eastern US, submissions were sometimes received from a broad and varied geographic range and few tested for pathogens or obtained epidemiological information from the patients including whether disease ensued [5, 6, 9, 12].

To better describe the potential public health implications of human tick attachment, the current study was conducted. The specific objectives were to 1) determine the geographic distribution and identity of ticks found attached to humans in Georgia, 2) test ticks for suspected or known zoonotic pathogens via polymerase chain reaction (PCR), 3) collect demographic, tick exposure, and bite risk data from tick submitters, and 4) determine whether any submitters became ill during the 3 weeks following the tick bite.

## Methods

### Collection of ticks and epidemiological data

From April 2005–December 2006, a state-wide media campaign was performed requesting that residents with a tick (s) attached to them contact the Georgia Poison Center (GPC) to enroll in the study. GPC staff answered any questions regarding tick removal and tick-borne disease, described the components of the study to all callers who met the inclusion criteria (i.e., persons who had an attached tick), obtained informed verbal consent, and provided tick shipping instructions to those who agreed to participate. They also collected basic contact information for the enrollee, date of tick removal, location of tick attachment on the body, and county in which the bite occurred.

Approximately 3 weeks later, a follow-up survey was administered by Georgia Division of Public Health (GDPH) staff during which the participant was asked to estimate how long the tick was attached, describe the setting where they were bitten, and report any illness since the tick bite. For those reporting any illness the following data was collected: onset date, duration of illness, first symptom, and yes/no to the following list of symptoms: fever, chills, nausea, rash, stomach pain, weakness/tiredness, muscle soreness, altered sense of taste, decreased appetite, painful to look at light or sun, dizziness, headache, diarrhea, joint pain, weight loss, vomiting, sweats, cough, confusion, and also asked if the patient experienced any other symptoms. For those who had a rash, they were asked where on the body it was located, whether it was at the site of the tick bite, and what it looked like. Patients were also asked whether they sought medical attention, had blood drawn, and/or was prescribed medicine for their illness.

In 2006 only, participants were asked about protective measures taken against ticks (both on purpose and not on purpose to avoid tick bites). Tick avoidance measures were divided into two categories, primary (e.g., things done to prevent ticks from attaching including, but not limited to, use of DEET or other repellant, keeping extremities covered by clothing, and/or not sitting on the ground) and secondary protective measures (actions performed to find and remove ticks once attached including checking oneself frequently for ticks while outside, full body and/or buddy tick checks, and taking a shower). This study was reviewed by a member of the Georgia Department of Human Resources Institutional Review Board and received a non-research determination.

### Tick identification and pathogen testing

Lab personnel responsible for identifying and testing ticks for pathogens were blinded to whether or not the tick bite victim developed illness. All ticks were classified to species, life stage and gender using published morphological keys [13, 14]. Ticks were then homogenized and stored in phosphate buffered saline (PBS) and stored at − 80 °C until testing. DNA was extracted from 100 μL of the homogenized tick solution using a Qiagen Viral RNA Minikit (Qiagen Science, Valencia, CA) as per the manufacturer’s instructions. Testing ticks for bacterial agents was performed using several previously published (Table 1) nested polymerase chain reaction (PCR) protocols which targeted the 16S rRNA genes of *E. chaffeensis* and *E. ewingii*, the 17 kDa gene of *Rickettsia* spp., the flagellin gene (*fla*) of *Borrelia* spp., and the fopA gene of *Francisella tularensis*. Ticks were also tested for Panola Mountain *Ehrlichia* and these data have been summarized previously [15].

**Table 1:** Polymerase chain reaction protocols used for testing ticks for selected pathogens

Pathogen	Gene Target/Reference	Primers	Annealing Temperature
			Primary/Secondary
*E. chaffeensis*	16S rRNA/[39]	Primary: ECC (5′-AGAACGAACGCTGGCGGCAAGCC)	55 °C/55 °C
		Primary: ECB (5′-CGTATTACCGCGGCTGCTGGCA)	
		Secondary: HE1 (5′-CAATTGCTTATAACCTTTTGGTTATAAAT)	
		Secondary: HE3 (5′-TATAGGTACCGTCATTATCTTCCCTAT)	
*E. ewingii*	16S rRNA/[39, 40]	Primary: ECC (5′-AGAACGAACGCTGGCGGCAAGCC)	55 °C/48 °C
		Primary: ECB (5′-CGTATTACCGCGGCTGCTGGCA-3′)	
		Secondary: HE3 (5′-TATAGGTACCGTCATTATCTTCCCTAT-3′)	
		Secondary: EE72 (5′-CAATTCCTAAATAGTCTCTGACTATT-3′)	
*Rickettsia* spp.	17-kDa antigen/[41, 42]	Primary: 17kD1 (5′-GCTCTTGCAACTTCTATGTT-3′)	48 °C/48 °C
		Primary: 17kD2 (5′-CATTGTTCGTCAGGTTGGCG-3′)	
		Secondary: 17 k-5 (5′-GCTTTACAAAATTCTAAAAACCATATA)	
		Secondary: 17 k-3 (5′-TGTCTATCAATTCACAACTTGCC)	
*Borrelia* spp.	Flagellin (*flaB*)/[43]	Primary: FLALL (5′-ACATATTCAGATGCAGACAGAGGT)	55 °C/55 °C
		Primary: FLARL (5′-GCAATCATAGCCATTGCAGATTGT)	
		Secondary: FLALS (5′-AACAGCTGAAGAGCTTGGAATG)	
		Secondary: FLARS (5′-CTTTGATCACTTATCATTCTAATAGC)	
*F. tularensis*	FopA/[44]	Primary: FNA8L (5′-CGAGGAGTCTCAATGTACTAAGGTTTGCCC)	55 °C/55 °C
		Primary: FNB2L (5′-CACCATTATCCTGGATATTACCAGTGTCAT)	
		Secondary: FNA7L (5′-CTTGAGTCTTATGTTTCGGCATGTGAATAG)	
		Secondary: FNB1L (5′-CCAACTAATTGGTTGTACTGTACAGCGAAG)	

For each primary reaction (except the Borrelia *fla* gene), the PCR reactions were assembled in 25 μL volumes containing 11 μL of molecular grade biological water (MGBW), 2.5 μL of MgCl_2_, 5 μL of GoTaq Flexi Clear Buffer (Promega, Madison, WI), 0.25 μL of dNTPs (20 mM initial concentration), 0.5 μL of each primer (40 μM initial concentration), 0.25 μL of GoTaq Flexi (Promega, Madison, WI), and 5 μL of extracted DNA. For the primary reaction targeting the *Borrelia fla* gene, the same volumes were utilized except, 10 μL of extracted DNA and 6 μL of MGBW were added to the reaction. For each secondary reaction, volumes of 25 μL were assembled containing 15 μL of MGBW, 2.5 μL of MgCl_2_, 5 μL of GoTaq Flexi Green Buffer (Promega, Madison, WI), 0.25 μL of dNTP, 0.5 μL of each primer, 0.25 μL of GoTaq Flexi, and 1 μL of the primary PCR product.

Amplified products were separated using gel electrophoresis on a 2.5 % agarose gel that was stained with ethidium bromide and visualized using a UV light. All amplicons for *Rickettsia* and *Borrelia* spp. were purified using a Qiagen gel extraction kit (Qiagen, Valencia, CA, USA) and bi-directionally sequenced at either the Integrated Biotechnology Lab at the University of Georgia (Athens, GA) or the Clemson University Genomics Institute (Clemson, SC). Resulting sequences were compared to published sequences in the GenBank database.

Precautions were taken to prevent and detect contamination including performance of primary and secondary reactions, and product analysis in distinct, designated areas. Negative controls were included in each DNA extraction and PCR reaction. Furthermore, positive controls were included in each set of PCR reactions and consisted of cultures of *E. chaffeensis*, *B. lonestari*, *F. tularensis* (LVS strain); a canine blood sample positive for *E. ewingii*; and a tick positive for *Rickettsia*.

### Statistical analysis

For purposes of statistical analysis, when applicable, *E. chaffeensis*, *E. ewingii*, Panola Mountain *Ehrlichia* and *R. parkeri* were considered pathogenic bacteria and all other *Rickettsia* species detected, other than *R. amblyommii*, were considered nonpathogenic bacteria. Note that *R. rickettsii and F. tularensis*, known pathogens, were not detected in this study. *Borrelia lonestari* and *R. amblyommii* were analyzed separately as agents of unknown pathogenicity status. Counties were designated as being in the Coastal, Coastal Plain, Mountain, or Piedmont georegion of the state. Fisher’s exact test of independence was used to evaluate the significance of associations between tick species and georegion or site of body attachment; use of tick bite prevention and gender or location at time of bite (i.e. at home or away from home); and type of tick species or tick-borne pathogen prevalence and owning or working with animals.

Fisher’s exact test of independence was also used to evaluate the univariate associations between predictor variables and the probability of developing any type of illness (this included illness with symptoms compatible with a tick-borne disease and otherwise). Predictors having a univariate *p*-value less than or equal to 0.2 were included in a maximum multivariable logistic regression model for the prediction of illness. Multivariable model selection proceeded by manual stepwise elimination from the maximum model until only variables with a *p*-value less than 0.05 remained. Variables considered in the univariate analysis include use of primary protection, use of secondary protection, bitten by tick positive for pathogenic bacteria, bitten by tick positive for non-pathogenic bacteria, bitten by tick positive for *R. amblyommii*, bitten by tick positive for *B. lonestari*, and time tick was attached. Variables that had a *p*-value less than or equal to 0.2 in this univariate analysis and therefore were considered in the multivariable analysis included whether the participant had a tick attached to them that was positive for a known pathogen, whether the person utilized secondary protective measures, and the amount of time the tick was attached to the participant.

## Results

**Fig. 1:**
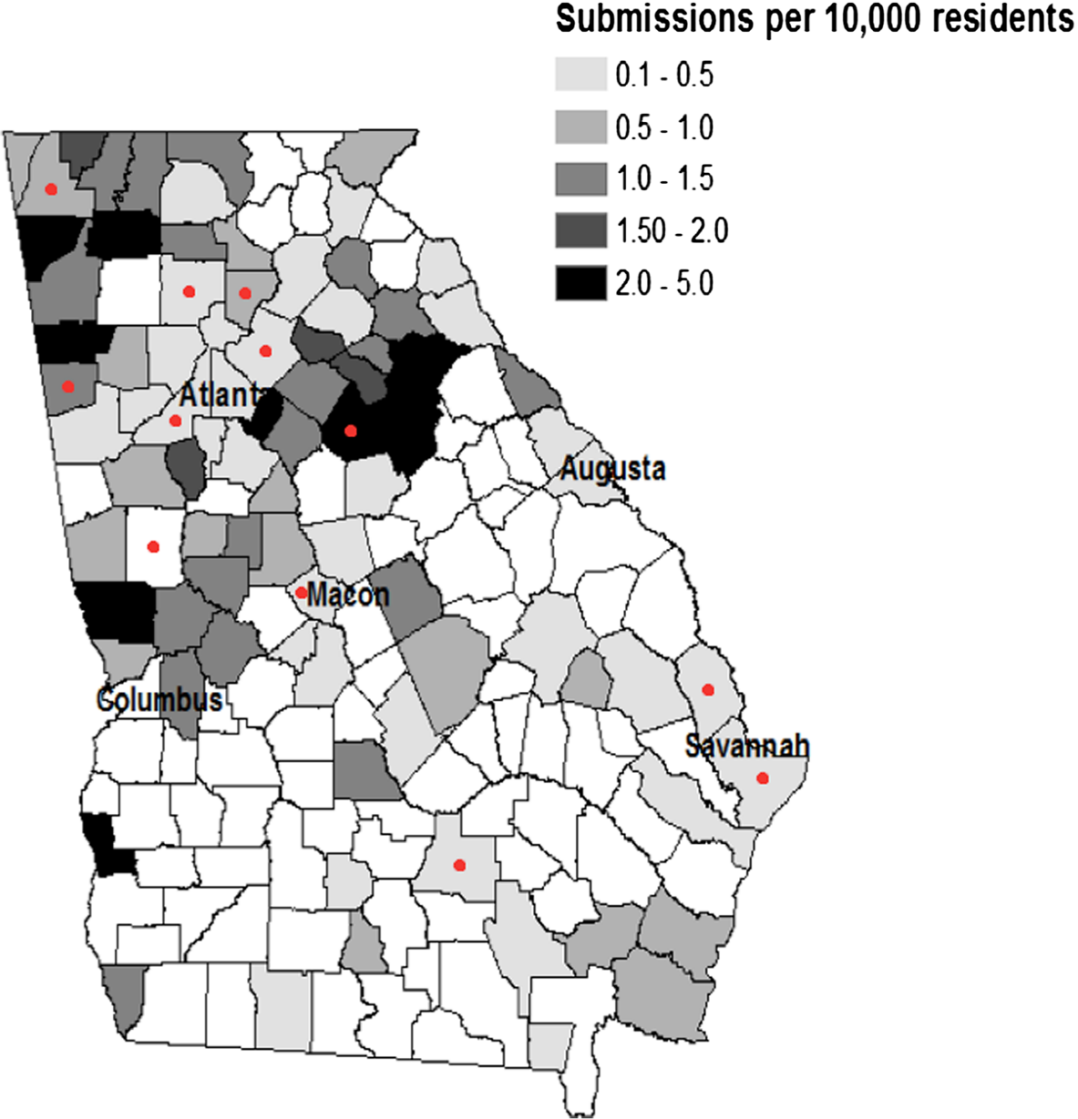
Georgia state map of total submissions per 10,000 residents by county. Note that map denotes participants’ resident county, not necessarily where the tick was acquired. Red dots indicate counties where ticks were acquired (not necessarily individual’s county of residence) that resulted in an illness that fit criteria for a potential tick-borne disease

A total of 597 ticks (426 *A*. *americanum*, 142 *D. variabilis*, 19 *A. maculatum*, 7 *I. scapularis*, and 3 *Amblyomma* sp.) (Table 2) were submitted by 444 participants (range of 1–17 ticks/person). These participants resided in 85 of the 159 (53 %) counties in Georgia and were bitten in 95 (60 %) counties (Fig. 1). Submissions by county of residence (although not necessarily where the tick was obtained) ranged between 0.08 and 4.60 ticks per 10,000 county residents with Greene, Clay, Morgan, Harris and Chattooga counties having the highest submission rates (4.60, 3.07, 3.00, 2.86, and 2.72 ticks per 10,000 residents respectively) (Fig. 1). In 2005, 483 ticks were submitted for which the date on which they were removed was recorded, with *D. variabilis* adults and *A. americanum* adults and nymphs peaking in May. Although this corresponded with peak enrollment in the study, seasonality was difficult to analyze because this also corresponds with the timing of our media advertisement of the project. Submission rates in 2006 were too low to evaluate seasonality of submissions with only 85 ticks being submitted for which date of removal was recorded.

**Table 2:** Summary of ticks collected by species, life stage, and polymerase chain reaction (PCR) assay results

Tick species	Stage	No.	*Ehrlichia chaffeensis*	*Ehrlichia ewingii*	*Ehrlichia* sp. PME	*Borrelia lonestari*	*Rickettsia* spp.	*Rickettsia* sp. Identity
			No. ticks positive (% total ticks)					
*Amblyomma americanum*	Adult^a^	241	1 (0.4)			2 (0.8)	76 (32)	*R. amblyommii* – 72 (30)
								*R. montana*–1 (0.4)
								*R. rhipicephali*-1 (0.4)
								*R. parkeri*–1 (0.4)
								*R*. sp. TR–39–1 (0.4)
	Nymph	185					46 (25)	*R. amblyommii*–45 (24)
								*R*. sp.–1 (0.5)
*Amblyomma maculatum*	Adult	18					4 (22)	*R. amblyommii*–1 (5)
								*R. parkeri*–1 (5)
								*R*. ARANHA–1 (5)
								*R*. sp.–1 (5)
	Nymph	1					1 (100)	*R. amblyommii*–1 (100)
*Amblyomma* sp.	Nymph	3					2 (67)	*R. amblyommii*–2 (67)
*Dermacentor variabilis*	Adult^a^	142		2 (1)	1 (0.7)		18 (13)	*R. amblyommii*–2 (1)
								*R*. ARANHA–1 (0.7)
								*R. montana*-15 (10)
*Ixodes scapularis*	Adult	7					6 (86)	*R*. sp. TR-39–4 (57)
								*R. cooleyi*–1 (14)
								*R*. sp. Is-1–1 (14)

^a^One was co-infected

**Fig. 2:**
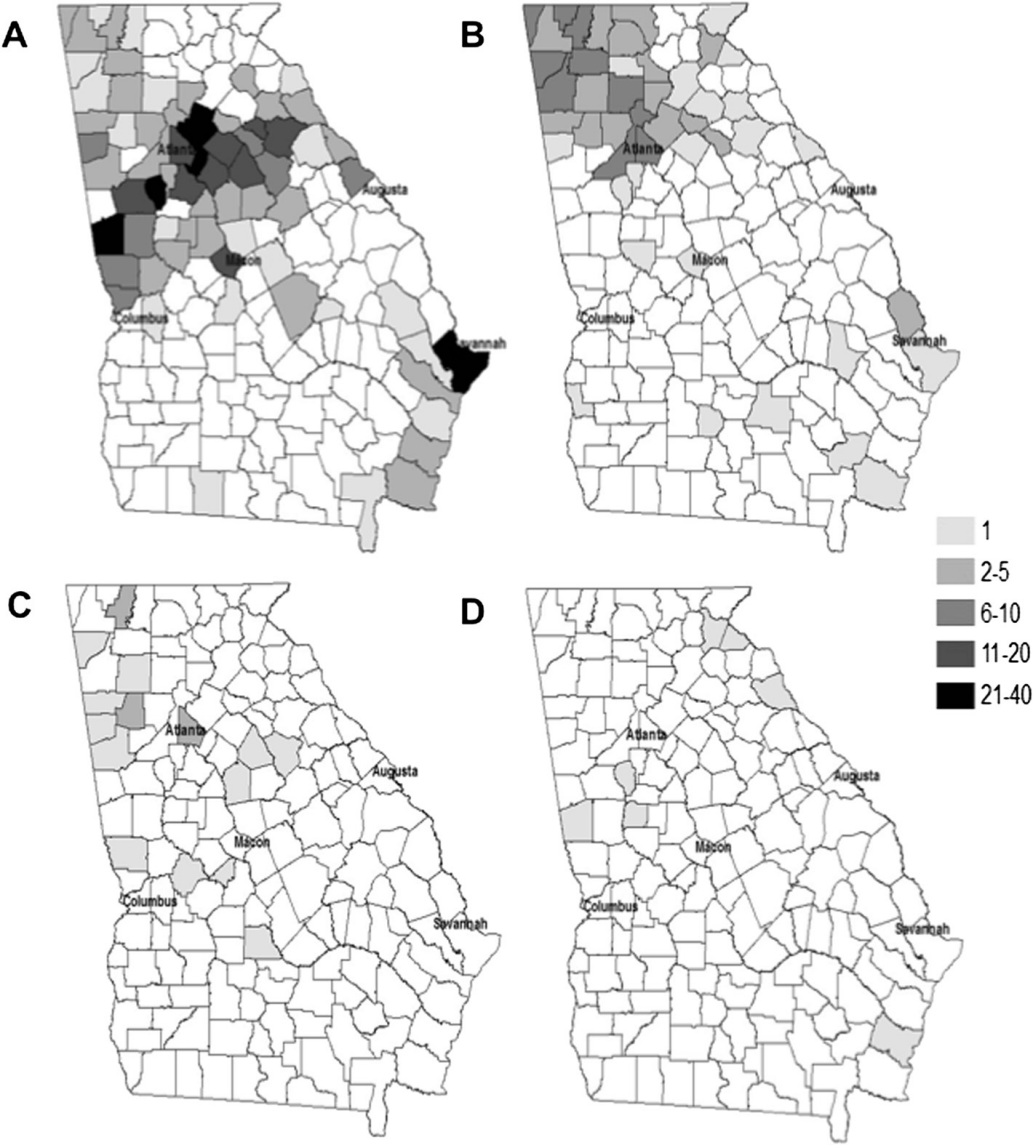
Maps with numbers of ticks by species originating from each county in Georgia. **a**. = *A. americanum*, **b**. = *D. variabilis*, **c**. = *A. maculatum*, and **d**. = *I. scapularis*

For those participants that provided demographic data, 209 were male, 203 were female and 399 were Caucasian, 8 were African American, 4 were multi-racial, and 1 was Asian. Georegion in which the tick was known to have attached to a participant was found to be significantly correlated with tick species (*p* < 0.001), with *A. americanum* being most commonly detected in the Piedmont and *D. variabilis* being commonly detected in the Mountain and Piedmont regions (Fig. 2a and b). *A. maculatum* and *I. scapularis* were submitted too infrequently to evaluate regional associations (Fig. 2c and d).

Of the ticks submitted, the location of attachment on the body for 534 was reported. The location of attachment and tick species was significantly associated (*p* < 0.001), with *A. americanum* primarily attaching to the trunk (52 %) and legs (32 %) followed by the head (11 %) and arms (5 %) and *D. variabilis* primarily attaching to the head (64 %), followed by the legs (17 %), trunk (15 %), and arms (4 %). Other tick species were not collected in large enough number to evaluate associations with attachment site, however, the majority of *A. maculatum* and *I. scapularis* were attached to the trunk (44 % and 43 %, respectively) and head (33 % and 29 %), followed by the legs (17 % and 14 %) and arms (6 % and 14 %).

Seventy-four participants were asked about protective measures taken against tick bites. Of these, 65 (88 %) used some type of protective measure  against ticks, although only 25 (34 %) took some type of protective measure (s) to purposely prevent tick bites and/or remove ticks once attached. Whether done on purpose or not, primary protective measures were taken by 55 (74 %) participants (12 specifically to avoid ticks) and secondary protective measures were taken by 59 (80 %) participants (25 to avoid ticks) while 49 participants (66 %) utilized both primary and secondary protective measures. Primary protective measures (done on purpose or not) were not found to significantly reduce the chance of illness after tick exposure (*p* = 0.29), while secondary protective measures did reduce the chance of illness (*p* = 0.04) (Table 3). Neither the participant’s gender nor location at time of bite (i.e., at or away from one’s home residence) was significantly associated with whether or not they purposely took preventative measures (*p* = 0.24 and *p* = 0.20 respectively).

**Table 3:** Associations between protective measures against tick bites and illness, gender, and proximity to home residence

	Illness After Tick Bite (%)			Gender (%)			Location When Bitten (%)			
	Sick	Not Sick	*p*-value ( vs. illness)	Male	Female	*p*-value (gender vs. )	Away from Home	At Home	Unknown	*p*-value (location vs. )
No Primary ^a^(*n* = 14)	5 (36)	9 (64)	0.29	9 (64)	5 (36)	0.52	2 (14)	11 (78)	1 (8)	0.18
Primary (*n* = 55)^b^	11 (20)	44 (80)		27 (49)	28 (51)		27 (49)	19 (35)	9 (16)	
No Secondary ^a^(*n* = 10)	5 (50)	5 (50)	0.04	4 (40)	6 (60)	0.19	5 (50)	5 (50)	0	0.4
Secondary Protection (*n* = 59)^b^	11 (19)	48 (81)		32 (54)	27 (46)		24 (41)	25 (42)	10 (17)	

^a^5 participants using neither primary nor secondary protection declined to answer questions related to illness, gender or location when bitten

^b^Note that 12 of 55 and 25 of 59 individuals took primary and secondary protection measures respectively

One hundred and forty participants had at least one tick attached to them that was PCR positive for one or more species of bacteria. Georegion was found to be significantly associated with the prevalence of non-pathogenic *Rickettsia* spp., with the Coastal region having the greatest prevalence (15 %) (*p* = 0.008) followed by the Coastal Plain (11 %), Mountain (10 %), and Piedmont (4 %) regions. While no other statistically significant associations between georegion and bacteria prevalence were found, the prevalence rates of *R. amblyommii* by region (Coastal, Coastal Plain, Mountain, and Piedmont respectively) were 33 %, 20 %, 16 %, and 25 % and for pathogenic bacteria were 4 %, 0, 1 %, and 1 %. A single tick infected with *B. lonestari* was detected in the Piedmont region. Two ticks were co-infected, one with *E. chaffeensis* and *R. amblyommii* and another with *E. ewingii* and *R. montana*. Furthermore, six participants had multiple ticks attached to them infected with *R. amblyommii*, one participant had multiple ticks infected with *R. montana*, one participant had one tick infected with *R. amblyommii* and another tick infected with *R. montana*, and one participant had one tick infected with *R. amblyommii* and another tick infected with *R*. sp. TR-39.

Fifty (18 %) of the 282 individuals with no PCR positive ticks reported some type of illness (not necessarily clinically compatible with a tick-borne illness) within 3 weeks after their tick bite (s) as compared to 27 (21 %) of 129 participants with PCR positive ticks. Illness status of 32 additional participants with no PCR positive ticks could not be determined. Participants with an attached tick positive for a known pathogen were significantly more likely to report an illness (3/5) (not necessarily clinically compatible with a tick-borne illness) compared with other participants (74/406) (*p* = 0.048) (Table 4). In addition, participants that had ticks attached to them which were positive for either non-pathogenic *Rickettsia* spp. (*n* = 22) or *R. amblyommii* (*n* = 100) were not significantly more likely to become sick as compared to all other participants (number sick/total: 73/389 and 58/311 respectively) (*p* = 1.00 for both). Specifically, 3 (60 %) of 5 individuals with ticks positive for a pathogenic bacteria became sick as compared to 4 (18 %) of 22 individuals with non-pathogenic *Rickettsia* spp., 19 (19 %) of 100 individuals with ticks positive for *R. amblyommii*, and 1 (50 %) of 2 individuals with ticks positive for *B. lonestari*.

**Table 4:** Description of participants who submitted a tick positive for a species of pathogenic bacteria

Participant #	Tick Species	Pathogen	Time Attached (hrs)	Incubation, if applicable (days)	Went to Doctor	Symptoms
1	*D. variabilis*	*E. ewingii*	unknown	4	yes	fever, rash, weakness, myalgia, joint pain, congested, earache, swollen & runny eyes
2	*D. variabilis*	*E. ewingii* (& *R. montana*)	6-12	<1	yes	tight neck
3	*D. variabilis*	Panola Mountain *Ehrlichia*	2-6	n/a	no	not sick
4	*A. americanum*	*E. chaffeensis* (& *R. amblyommi*)	1-4	13	no	nausea, fever, chills, stomach pain, weakness, headache, joint pain, sweats, cough
5	*A. maculatum*	*R. parkeri*	unknown	n/a	no	not sick

Two participants receiving medical attention after the reported tick bite (not necessarily due to illness) tested positive for RMSF via serologic testing. Of those, one was exhibiting fever and vomiting and reported an IgM titer of 1:64 and an IgG of 0. However, the patient also reported having been previously diagnosed with RMSF via a blood assay, with the date of this diagnosis being unknown. Furthermore, this patient had submitted an *A. americanum* adult which was PCR negative. The second participant reporting RMSF positive results had submitted a single *D. variabilis* adult testing positive for *R. montana*. The patient reported having had no symptoms but had been tested as a pre-cautionary measure by their physician after the tick bite. The patient reported having an IgM of 0 and an IgG of 1:64, with no prior history of RMSF.

Participants who reported an illness were asked if they had been previously diagnosed with a tick-borne disease and eight participants indicated they had a previous diagnosis, of which, three were presumptively diagnosed by a physician (all with Lyme disease) and five were reportedly confirmed with a diagnostic assay (three for RMSF, one for HME, and one for Lyme disease). No additional information was collected regarding these previous diagnoses.

Thirty-two participants sought medical attention due to their illness. Nine had blood drawn and 23 were prescribed some type of medication. Interestingly, an additional seven participants went to a physician as either a precautionary measure and/or to have the tick removed, or for an unrelated reason and reported being prescribed preventive antibiotics for a tick-borne illness despite a lack of symptoms or laboratory findings. Furthermore, three other participants exhibited no symptoms, but self-medicated with antibiotics.

In the univariate analysis, duration of tick attachment, use of secondary , and presence of pathogenic bacteria were all significantly associated with illness (Table 5). No other variables in the univariate analysis had a *p*-value less than 0.20. Upon performing a multivariate analysis with logistic regression, duration of attachment was found to be the only variable significantly associated with development of illness (*p* = 0.037). Compared to patients that had ticks attached for less than 12 h, those with ticks attached for 12–24 h (OR [95 % CI] = 2.0 [1.0, 4.0]) and for 24–48 h (2.9 [1.3, 6.5]) were significantly more likely to become sick, while those with ticks attached for > 48 h (1.5 [0.6, 3.9]) were not significantly more likely to report a illness.

**Table 5:** Univariate analysis of potential risk factors for non-specific illness after a tick bite

Variable	*n*	# Sick (%)	^a^ *P*
Time Attached (hrs)			0.032
1-12	196	26 (13.3)	
12-24	68	16 (23.5)	
24-48	39	12 (30.8)	
>48	33	6 (18.2)	
Secondary			0.045
yes	59	11 (18.6)	
no	10	5 (50.0)	
Pathogenic Bacteria Positive			0.048
yes	5	3 (60.0)	
no	406	74 (18.2)	

^a^Fisher’s exact test of independence

Participants with missing information were excluded from statistical comparisons

The final diagnoses of patients seeking medical attention due to illness were not recorded in this study. However, to better determine whether any of the reported illnesses may have been a tick-borne disease, the data of the 27 participants who reported an illness and had a PCR positive tick attached to them were evaluated to determine if the illness fit the criteria of a potential tick-borne illness. Criteria to be considered a potential tick-borne illness included attached for longer than 6 h (or length of attachment was unknown), incubation period a minimum of 4 days (to exclude rash development or severe reactions to tick bites which could occur prior to 4 days), and the individual exhibited at least one of the following symptoms or signs: fever, chills, nausea, rash, stomach pain, weakness, myalgia, appetite loss, dizziness, headache, diarrhea, weight loss, vomiting, and/or sweats but did not have a cough, sore throat, or sinus congestion. Twelve of the 27 individuals evaluated exhibited symptoms that could potentially be related to a tick-borne illness (Table 6). No single county in Georgia had more than one case fitting criteria as a potential tick-borne illness (Fig. 1). Of these individuals, five sought out medical attention and of those, two had their blood drawn (the results or identity of blood test/s were not obtained for this study). However, the two participants receiving medical attention who had blood drawn were treated with doxycycline and tetracycline respectively. The remaining three seeking medical treatment were treated with clarithromycin, doxycycline, or a topical cream respectively.

**Table 6:** Description of participants meeting criteria for having a potential tick-borne illness

Patient	Time Attached (hrs)	Incubation (days)	Days Sick	Symptoms^a^	Went to Doctor/Blood Drawn	Medicine Prescribed (if any)	Species (stage)	Engorged	Pathogen (s)
2	6-12	0	2	**tight neck**	yes/no	clarithromycin	*D. variabilis* (adult)	no	*E. ewingii & R. montana*
6	unknown	6	unknown	**lump in neck**, joint pain	no	none	*D. variabilis* (adult)	partially	*R. montana*
7	6-12	12	1	**fatigue**, chills, nausea, stomach pain, weakness, muscles, loss of appetite, dizziness, headache, joint pain	no	none	*A. americanum* (adult)	no	*R. rhipicephali*
8	>48	11	2	**diarrhea**, stomach pain, weakness	no	none	*I. scapularis* (adult)	partially	*Rickettsia* sp. TR-39
9	unknown	unknown	14	**fever**, **myalgia**, rash, weakness, dizziness, headache, joint pain, bull’s-eye rash (grew to 13–15 cm diameter)	yes/no	doxycycline	7 *A. americanum* (nymphs)	no	1 *R. amblyommii*
10	24-48	5	21	**fatigue** fever, nausea, rash, weakness, myalgia, dizziness, headache, raised, reddish, swollen rash	yes/yes	doxycycline	7 *A. americanum* (nymphs)	no	4 *R. amblyommii*
11	>48	17	6	**headache**, nausea, weakness, loss of appetite	no	none	2 *A. americanum* (adult and nymph)	no	1 *R. amblyommii*
12	24-48	1	14	**rash**, joint pain, red 3 cm rash	yes/no	unknown topical cream	2 *A. americanum* (nymphs)	no	1 *R. amblyommii*
13	24-48	5	7	**insomnia**, chills, stomach pain, weakness, loss of appetite, headache, joint pain, sweats	yes/yes	tetracycline	*Amblyomma* sp. (unknown)	unknown	R. amblyommii
14	12-24	26	2	**fever**, chills, nausea, stomach pain, weakness, myalgia, loss of appetite, sweats	no	none	*D. variabilis* (adult)	partially	*R. amblyommii*
15	unknown	15	unknown	**pain in neck**	no	none	*A. americanum* (adult)	no	*R. amblyommii*
16	24-48	4	unknown	**sore eyes**, **extreme fatigue**, **fever**, nausea, rash, weakness, myalgia, photosensitivity, headache, diarrhea, joint pain, confusion, bulls-eye rash	no	none	*A. americanum* (adult)	no	*R. amblyommii*

^a^first symptom (s) in bold

Interestingly, two of the individuals with *R. amblyommii*-positive ticks who reported symptoms consistent with a potential tick-borne illness also reported a bulls-eye rash at the tick bite site. However, in one case, multiple ticks were submitted by the participant and it could not be determined whether the *R. amblyommii*-positive tick was located at the tick bite site around which the rash occurred.

Finally, data were collected regarding interactions with domestic animals. Four hundred and twelve participants owned dogs and 73 owned cats. One hundred and sixty-four dog owners (39.8 %) and 15 cat owners (20 %) allowed their dog/cat in the home. When asked if anti-tick medication had been used on their pets in the past 30 days, 27 cat owners (38 %) (10 of which were allowed in the home) and 162 dog owners (39.3 %) (126 of whom allowed their dog/s in the house) replied yes. It was also found that 42 (*n* = 412) worked with animals. Presence of dogs and/or cats in the home, use of anti-tick medication, and regular interaction with work animals, was not associated with type of tick species or pathogen prevalence.

## Discussion

This study is one of the most comprehensive epidemiologic studies to date on human tick bites and associated pathogens. In addition to providing information related to tick distribution in the state of Georgia, it provided a rare opportunity to follow individuals bitten by bacteria-positive ticks to determine whether a potential tick-borne illness developed. Furthermore, it provided insights into how and if potential tick-borne illnesses were diagnosed and/or treated. Additional information regarding protective measures used by the general public and risk factors for illness which could be used to better target and better educate the general public on tick-borne disease prevention was also collected.

This study collected information related to distribution of tick species at the county level throughout Georgia and in particular provides new county-level data on *A. maculatum* distribution which has sparse data associated with its distribution. It is of interest that prevalence of non-pathogenic *Rickettsia* was significantly higher in the Coastal region, which was not associated with the submission rate of its primary vector, *A. americanum* which was more common in the Piedmont region. This possibly indicates that *A. americanum* were equally or more abundant in the Coastal region as compared to the Piedmont region and that our study did not detect this due to higher submission rates from the more densely populated Piedmont region.

Individuals with ticks attached to them for greater than 12 h were more likely to develop non-specific illness. Although the illnesses may or may not have been tick-borne, most tick-borne pathogens require 12–24 h (and in some cases longer) to be transmitted [3, 16, 17]. Furthermore, the finding that some individuals either self-medicated and/or were put on antibiotics by a physician despite no laboratory and/or clinical symptoms is concerning for several reasons. While prophylactic treatment of tick bite victims is recommended in areas in which Lyme disease is hyperendemic [18, 19], the use of prophylactic antibiotic treatments in a state such as Georgia where Lyme disease is not hyperendemic raises concerns due to, among other things, increased reports of antibiotic resistant bacteria (unrelated to tick-borne bacteria) [20–22]. Furthermore, individuals whom are self-medicating are of particular concern due to the risk of taking the wrong type, dosage and/or duration of medication. These findings indicate that an effort to better educate both the general public and physicians regarding appropriate prophylactic treatment for tick bites is needed. Particularly in the case of physicians, they must have knowledge of tick-borne pathogens in their region and if prophylactic treatment would be recommended [23, 24].

While it is generally accepted that tick-borne disease incidence rates are higher than actually reported, the cause (s) of underreporting are unclear but are likely due to one or more factors such as not seeking medical treatment, misdiagnosis, lack of specific diagnostics, and not reporting confirmed cases [25, 26]. Overall, our study found that in individuals bitten by a tick, 2.7 % (*n* = 12) developed a possible tick-borne disease. Interestingly, there were only two instances in our study in which the participant stated that their physician reported their case as a tick-borne disease to the state health department, neither of which fit our criteria in this paper for a potential tick-borne disease. Furthermore, of the twelve participants who fit our criteria for a tick-borne illness, only five sought medical attention and of those, only two had their blood drawn. In addition to this, of the 5 individuals receiving medical attention, 2 were prescribed doxycycline, 1 tetracycline, 1 clarithromycin, and 1 a topical cream. Although our study relied on self-reported illness without definitive diagnoses and was biased due to the fact that participants a) found the ticks attached to them (versus having not), and b) perhaps had better overall awareness of tick-borne diseases as evidenced by them participating in the study, it still provides insight into the possible extent of failure to seek medical attention, misdiagnosis and underdiagnosis of tick-borne diseases.

In addition to gaining insight into under-and misdiagnosis of tick-borne diseases, this prospective study also allowed us to follow individuals bitten by ticks positive for bacteria that are of unknown pathogenicity such as *Borrelia lonestari* (originally suspected as the causative agent of STARI) [27, 28] and *Rickettsia amblyommii* (which has been questioned as causing rickettsiosis in some individuals) [29]. While *B. lonestari* was not associated with any of the individuals fitting the criteria for a potential tick-borne disease, 8 of our 12 cases fitting our criteria for a possible tick-borne disease had an *R. amblyommii*-infected tick attached, and 2 of these cases developed erythema migrans. A previous study [29] highlighted *R. amblyommii* as a potential cause of disease in humans and since then, Billeter et al. [30] reported a single case in which *R. amblyommii* was directly associated with a tick bite resulting in a rash. These observations appear to indicate that further research is warranted regarding the potential pathogenicity of *R. amblyommii*.

Prevention measures are critical to decrease the risk of tick bites and agencies have numerous recommendations for preventing exposure [31–33]. Our study found no association between gender and the use of preventive measures and furthermore, only 34 % of participants in the study knowingly used prevention measures. Although we suspect that this percentage may have been high due to the fact that the population sampled in the current study was cognizant enough of tick-borne illnesses to find the ticks and submit them, this percentage is similar to the findings of a nationwide study which mainly focused on primary protective measures [34]. Furthermore, similar to a study in the Netherlands [35], we found that of the individuals knowingly utilizing protective measures, secondary prevention measures such as tick checks were more commonly utilized than primary prevention measures.

Interestingly, our study indicates that the majority of our participants utilizing protective measures were doing so unknowingly. Because such a large percentage of individuals were unknowingly utilizing some type of preventative measure, better awareness and education could lead to more types of preventative measures being taken and/or more effective use of preventative measures (note that this study did not allow us to evaluate the effectiveness of the preventative measures taken due to the fact that we did not have control individuals). Indeed, it has been shown that primary and secondary prevention educational programs significantly reduce the incidence of tick-borne disease in targeted populations and/or increase the utilization of some types of prevention methods such as performing regular tick checks [36, 37].

Our study also found that sites of body attachment were significantly associated with tick species. These observations corroborate the findings of Felz and Durden [38] who also found that *D. variabilis* preferred attachment sites on the head, while *A. americanum* preferred the trunk and extremities. Knowing which parts of the body different tick species tend to attach to in conjunction with tick distribution and activity periods may allow for more effective tick inspection.

## Conclusions

Several important findings are reported in this study related to tick bite prevention, risk of human tick-borne pathogen exposure, and the epidemiology of human tick bites in Georgia. Identifying individuals fitting criteria for a possible tick-borne illness while finding others whose physicians were potentially incorrectly reporting a tick-borne disease provides evidence towards the common assumption that individuals with tick-borne diseases fail to seek medical treatment, are misdiagnosed, or underdiagnosed. Furthermore, our data provide valuable reports of tick species occurrence in Georgia. Collectively, these observations in conjunction with our findings regarding tick attachment site preferences by tick species and personal protective measures used against tick bites provide information that can be used for better public and physician education on tick bite prevention and tick-borne disease diagnosis. Specifically, these data indicate that the general public do use preventative measures but rarely for the purpose of avoiding ticks. To improve diagnosis of tick-borne diseases, better educating physicians on common tick-borne diseases in their region including clinical presentation, appropriate diagnostics, and treatments is necessary. Our study did not include testing of all ill participants and relied on the participants’ physicians to determine whether and what testing was appropriate for their illness. Thus we cannot determine whether or not self-reported illnesses were indeed tick-related. More detailed follow-up studies are recommended to further investigate the risk of developing a tick-borne disease following a tick bite with both pathogen-positive ticks as well as *R. amblyommii* positive ticks.
